# The breaking of fungal spore dormancy: A coordinated transition

**DOI:** 10.1371/journal.pbio.3002077

**Published:** 2023-04-21

**Authors:** Sjoerd Johan Seekles

**Affiliations:** Department of Molecular and Cellular Biology, University of Geneva, Geneva, Switzerland

## Abstract

The transition from dormant fungal spore to germling has been topic of study and debate. This Primer explores a recent discovery in PLOS Biology showing that chaperone Hsp42 plays a crucial role in re-solubilizing the proteome during dormancy breaking, although a role of trehalose cannot be excluded.

## Teaser

The transition from dormant spore to germling has been topic of study and debate. A recent discovery in *PLOS Biology* shows that chaperone Hsp42 plays a crucial role in resolubilizing the proteome during dormancy breaking, although a role of trehalose cannot be excluded.

Fungal spores are stress-resistant cell types that can survive extreme conditions, such as high temperatures and high exposure to UV radiation [1]. The high stress resistance of fungal spores allows them to survive our preventive treatments and subsequently cause food spoilage and fungal infections [2]. Moreover, fungal spores are dormant, metabolically (nearly) inactive, and can stay viable in this state even after 17 years of storage [3].

Fungal spores accumulate high concentrations of compatible solutes and small protective proteins to ensure their survival. Total internal compatible solute concentrations of 1M have been reported [1]. This high concentration of compatible solutes and proteins causes the fungal spore’s cytoplasm to be highly viscous. Besides generating a high stress resistance, this increased viscosity arrests the cell’s metabolic activity. The spore’s cytoplasm containing high trehalose concentrations has been described as a “glassy state” where molecules, in particular proteins, are no longer freely moving [4]. This state causes proteins to be nonflexible and could affect proper folding of proteins [5]. As such, enzymes, although preserved, are thought to be mostly inactive when inside this glassy state.

**Fig 1:**
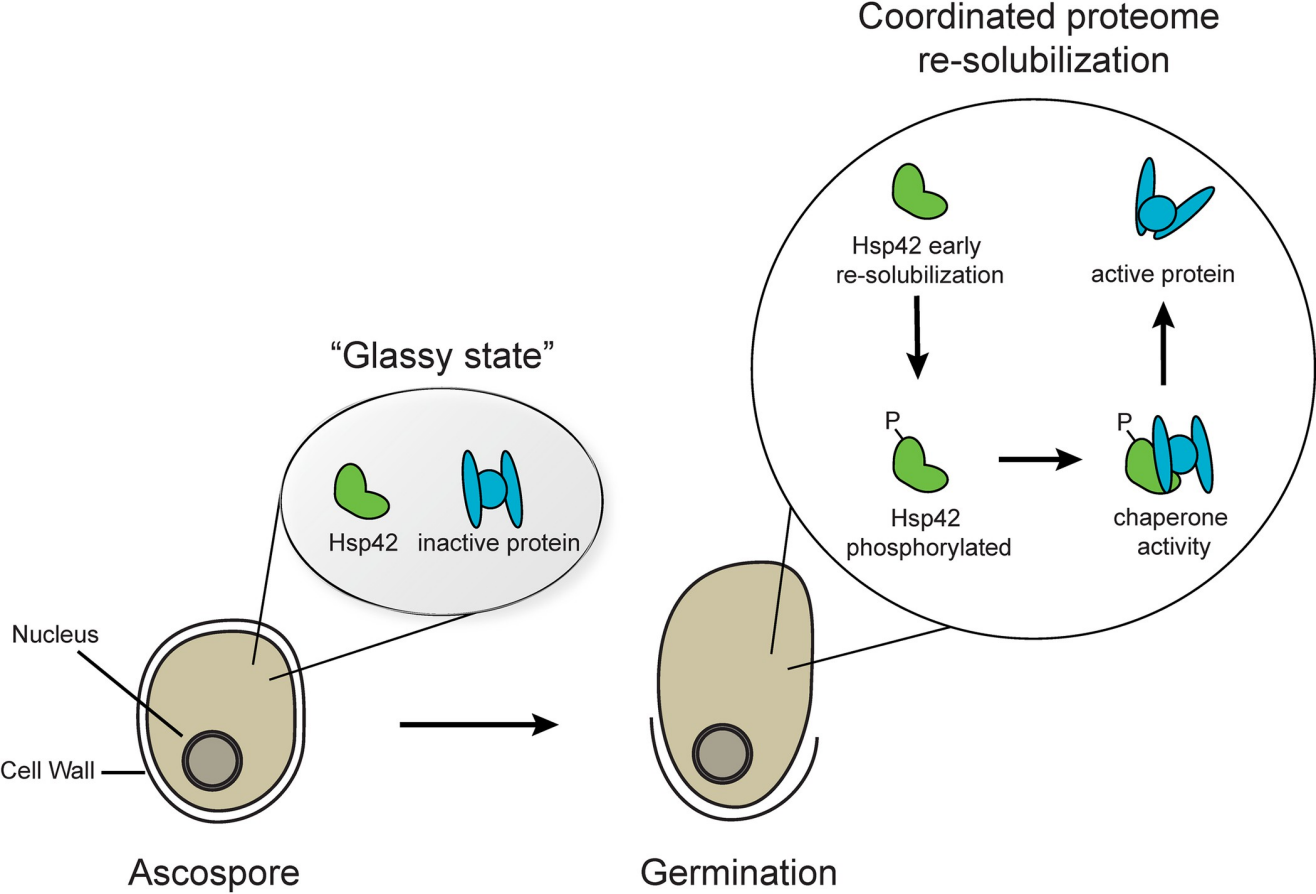
Protein resolubilization during germination is at least in part orchestrated by chaperone Hsp42. A cartoon visualizing protein resolubilization and the function of chaperone Hsp42 during this transition. Metabolically (nearly) inactive fungal spores contain a highly viscous cytoplasm. The highly viscous cytoplasm resembles a “glassy state” in which proteins are preserved and inactive. During germination, the viscous cytoplasm transitions to a watery cytoplasm. Some proteins resolubilize earlier than others, one such protein is chaperone Hsp42. During early stages of germination, Hsp42 gets phosphorylated and, therefore, activated and ensures proper refolding and resolubilization of the spore’s proteome.

How do these dormant spores with limited metabolic activity transition from this nearly inactive state to a lively germling? A recent study published in *PLOS Biology* by Plante and colleagues shows that the proper transition is at least in part orchestrated by chaperones, specifically Hsp42 [6]. The authors show that this heat shock protein is activated by phosphorylation during very early stages of germination and ensures proper resolubilization of proteins during breaking of dormancy. Strains lacking Hsp42 germinate slower and show a slower transition from the highly viscous condition to a cytoplasm, which is more fluid. This could be in part due to higher trehalose concentrations, which Plante and colleagues show remain higher during the dormancy breaking of *hsp42*Δ spores when compared to wild-type spores. Crucially, however, mutant strains containing a Hsp42 protein with a nonphosphorylatable amino acid residue, which causes the protein to no longer be activated during germination, show slower germination rates. Therefore, it can be concluded that the resolubilization of the cytoplasm during early stages of germination is at least in part orchestrated by the chaperone Hsp42 (Fig 1).

The novel role for Hsp42 during germination as described by Plante and colleagues is the first report, to my knowledge, to show that spores actively ensure and even coordinate proper reentering of the metabolically active state. As described by Plante and colleagues, proteins have different solubility levels, meaning that certain proteins will regain their native function before others. This finding gives new insights into the germination process and reveals the importance of the state proteins are in during germination, not merely their presence or abundance.

Interestingly, the findings of Plante and colleagues were driven by the hypothesis that a dormant spore is a stress response state and that germination can be regarded as a form of stress relief. Indeed, dormant spores do share characteristics with vegetative cells entering a stress response state. Plante and colleagues show a lowered pH and lowered movement of particles inside budding yeast ascospores when compared to vegetative cells. This increase in cellular acidity and viscosity is in line with a vegetative cell’s stress response and has been shown before [7]. Additionally, we know that fungal spores accumulate small protective proteins and compatible solutes, again showing similarities with a stress response. The model of fungal spores being in a constant stress response state is an interesting hypothesis and proofed a useful comparison in this study.

This exciting new discovery opens the door for future research directions and reevaluation of existing data. For example, it has been reported in several transcriptome and proteome studies that dormant fungal spores contain high concentrations of chaperones [8–10]. The study presented by Plante and colleagues shows a novel role for these proteins in spores, not solely as protectant for the dormant spore per se, but to ensure proper resolubilization and refolding of the proteome upon germination. Future research could focus on potential other chaperones involved in proteome resolubilization and whether this phenomenon is yeast ascospore specific. Additionally, the potential role of trehalose in the protein resolubilization needs to be further investigated, as this disaccharide is accumulated in many fungal spore types and is considered one of the main reasons for the spore’s viscous cytoplasm.
